# Knowledge of Essential Newborn Care and Associated Factors among Nurses and Midwives: A Cross-Sectional Study at Public Health Facilities in Wolaita Zone, Southern Ethiopia, 2019

**DOI:** 10.1155/2020/3647309

**Published:** 2020-03-18

**Authors:** Aseb Arba, Zerihun Zana

**Affiliations:** ^1^Wolaita Sodo University, College of Medicine and Health Sciences, Department of Nursing, Ethiopia; ^2^Wolaita Zone Health Department, Ethiopia

## Abstract

**Background:**

Knowledge of essential newborn care and proper practice is important for the survival, growth, and development of a newborn. In spite of its essentiality, most health-care professionals do not know and follow the World Health Organization recommendation. Therefore, this study is aimed at assessing knowledge of essential newborn care and associated factors among nurses and midwives working in maternal health case team at public health facilities of Wolaita Zone, Ethiopia, 2019.

**Methods:**

Institution-based cross-sectional study design was conducted from March to April 2019. Data were collected by using pretested questionnaire, and 36 public health facilities were selected after stratifying them based on their level of service and number of nurses and midwives working in maternal health-care team. All 218 nurses and midwives who were working in the delivery unit from selected facilities were included in the study. The collected data were entered into Epi data 3.02 and exported to statistical software for social sciences version 22 for analysis. Descriptive, bivariate, and multivariate analyses were done. Statistical significance of variables was declared as a *p* value < 0.05, and strength of association was adjusted odds ratio at 95% confidence interval in the final model.

**Result:**

A total of 218 nurses and midwives were participated in the study. Among them, 57.9% of participants had good knowledge of essential newborn care. The type of profession (AOR = 5.79, [2.47, 13.58]), educational level (AOR = 3.26, [1.42, 7.52]), interest to work in delivery room (AOR = 4.85, [1.89, 12.42]), and presence of guidelines (AOR = 2.29, [1.18, 4.45]) were the factors significantly associated with having knowledge of essential newborn care. *Conclusion and Recommendation*. The nurses and midwives had poor knowledge of some components of essential newborn care in the study area. Bachelor level of study, interest to work in delivery room, and being a midwife were the factors independently associated with knowledge of essential newborn care among nurses and midwives. Therefore, the head of labor ward and institution, zonal and woreda health units, and nongovernmental organizations who are working on maternal and child health should work on providing continuous education, providing incentives and motivators to improve interest to work in delivery unit, and providing guidelines in the unit.

## 1. Introduction

Birth is a major challenge to the newborn to negotiate intrauterine to extrauterine life successfully. The transition from intrauterine to extrauterine life is a dramatic one and demands considerable and effective physiological alterations by the baby in order to ensure survival [1, 2]. This immediate care time is crucial to the babies for subsequent well-being and adaptation. Skilled care during labor and childbirth with prompt management of complications alone can prevent about 50% of newborn mortality. Continuation of adequate newborn care in the postnatal period can prevent 75% of current newborn deaths [3].

The essential newborn protocol is a series of time bound and chronologically ordered care that a baby receives at birth, and it has standardized effective procedural steps: dry and stimulate, evaluate breathing, cord care, keep the newborn warm, initiate breastfeeding within the first one hour, administer eye drops/eye ointment to prevent eye infection, administer vitamin K intramuscularly, place the newborn's identification bands, weigh the newborn when it is stable and warm, and record all observations and treatment provided [1, 4–6].

Globally, 2.5 million children died in the first month of life at an average rate of 18 deaths per 1,000 live births in 2017. There are approximately 7,000 newborn deaths every day, accounting for about 47% of all child deaths. Globally, the number of neonatal deaths declined from 5 million in 1990 to 2.5 million in 2017 [7]. Promotion of ENC is one strategy for improving newborn health outcomes, but the standardized procedure for providing ENC is not commonly practiced [8].

The reason of neonatal death in Uganda was the low levels of knowledge among health workers regarding newborn care [9]. Overall average knowledge score on essential newborn care in India was 78% [10]. In Africa, 79% care providers dried baby with towel, 94% care providers cut cord with clean blade, 43% care providers initiated breastfeeding within the first one hour, 45% care providers placed new born skin to skin contact, and 65% clamped cord when pulsation stop or 2-3 min after birth [11]. Study conducted in Sudan Khartoum showing knowledge of the immediate care of the newborn showed that knowledge level was medium, that is, 50.6% [12]. Health professional's knowledge of the dosage of vitamin K was poorly known by the staff, that is, 18.9% for midwives and 17.2% for nurses [13].

A study conducted in Jimma zone on knowledge and practice of essential newborn care among nurses and midwives showed that 47.8% of respondents had poor knowledge of essential newborn care and on some components of essential newborn care [3, 14]. Other study conducted in Tigray central zone on knowledge and practice of immediate newborn care among midwives showed that one-fourth of respondents had poor knowledge of essential newborn care. A study conducted in Bahir Dar city on knowledge and practice of immediate newborn care among health professionals showed that 13.1% of respondents had poor knowledge of essential newborn care [2].

United Nations planned to end the preventable deaths of newborns from 22 deaths per 1,000 live births to 12 deaths per 1,000 live births by 2030 in sustainable development goal 3 [15]. The current Ethiopian situation of newborn mortality is far away from UN targets. To fill this gap, the Ethiopian government in collaboration with other partners has launched various initiatives for advancing knowledge and skill of health-care providers through training health-care professionals, providing basic equipment, and drugs and community education through different media about antenatal care follow-up and institutional delivery [16].

Despite implementation of different strategies to reduce neonatal mortality rate, it was remained stable at 37 deaths per 1,000 live births in 2011 and 29 deaths per 1,000 live births in 2016 according to Ethiopia Demographic and Health Survey (EDHS) [17]. Wolaita Zone is one of the zones in Ethiopia where neonatal sepsis and death are frequently occurring. Knowledge of ENC is basic to provide essential newborn care and reduce neonatal death. Therefore, this study was aimed at assessing the knowledge of health care provides on essential newborn care and recommending solutions on the identified gaps to the health facility heads, health system managers, program managers, and program implementers.

## 2. Objective of the Study

1. To assess knowledge of essential newborn care among nurses and midwives working in maternal health case team at public health facilities of Wolaita Zone, 2019
2. To identify factors associated with knowledge of essential newborn care among nurses and midwives working in maternal health-care team at public health facilities of Wolaita Zone, 2019

## 3. Methods and Materials

### 3.1. Study Area

The study was conducted in public health facilities of Wolaita Zone. Wolaita Zone is one of the 14 zones in Southern Peoples' Region of Ethiopia. The capital city of the zone is about 327 km far away from Addis Ababa. In the zone, there were 5 governmental hospitals, 2 nongovernmental hospitals, 37 private clinics, and 68 health centers. There were 406 midwives and nurses who were working in MCH case team at public health facilities of Wolaita Zone. Among them, 324 were midwives and 82 are nurses.

### 3.2. Study Design and Period

We used institution-based cross-sectional study from March to April 2019.

### 3.3. Source Population and Study Population

#### 3.3.1. Source Population

All nurses and midwives who were working in maternal health case team at public health facilities of Wolaita Zone from March to April 2019 were the source population.

#### 3.3.2. Study Population

All nurses and midwives who were working in delivery and postnatal care unit and fulfilled the inclusion criteria in selected public health facilities of Wolaita Zone during data collection period were the study population.

#### 3.3.3. Study Unit

A nurse or midwife who was working in labor, delivery, and immediate postnatal care service in selected public health facilities of Wolaita Zone during study period was taken as a study unit.

### 3.4. Inclusion and Exclusion Criteria

#### 3.4.1. Inclusion Criteria

- All midwives and nurses who were working in labor, delivery, and immediate postnatal unit in public health facilities at the time of data collection were included in the study.

#### 3.4.2. Exclusion Criteria

- Nurses and midwives who were in annual leave and students were excluded from the study.

### 3.5. Sample Size Determination and Sampling Technique

#### 3.5.1. Sample Size Determination

Sample size was calculated using single population and double population formula to obtain maximum sample size. After adding nonresponse rate, the final sample size determined was 218. So, it was planned to collect data from 218 health professionals that work in labor, delivery, and immediate postnatal care units in public health facilities.

#### 3.5.2. Sampling Procedure

Public health facilities were stratified based on the level of service they provide and the number of professionals working in labor, delivery, and immediate postnatal ward. From that, one teaching and referral hospital, four primary hospitals, and 31 health centers were selected. Finally, all nurses and midwives who were working in labor and delivery unit in selected public health facilities of Wolaita Zone were enrolled in the study. Totally, 218 participants were enrolled in the study. Among these, 16 participants were from one referral hospital, 40 participants were from four primary hospitals, and 162 participants were from 31 health centers.

### 3.6. Study Variables

#### 3.6.1. Dependent Variables

- Knowledge of essential newborn care

#### 3.6.2. Independent Variables

- Sociodemographic and socioeconomic factors
- Personal factors
- Institutional factors

### 3.7. Operational Definition and Definition of Terms

*Good knowledge*: when the health-care provider answered the knowledge question above or equal to the mean, he/she was considered as having a good knowledge.

*Poor knowledge*: when the health-care provider answered below mean in the knowledge questions, he/she was considered as having a poor knowledge.

*Essential newborn care*: the care provided to a newborn immediately after delivery which includes the time between births to 24 hours care is essential newborn care, and it includes to dry and stimulate, evaluate breathing, keep the newborn warm (prevent hypothermia), initiate breastfeeding in the first one hour, administer eye ointment, administer vitamin K intramuscularly, weigh the newborn when it is stable and warm, cord care, and delayed bathing of the baby for 24 hours after birth [18].

### 3.8. Data Collection Instruments

Data were collected through self-administered questionnaire which was used to assess knowledge of health-care providers on essential newborn care. Questionnaire was adapted from different literatures, and the WHO guidelines were used to collect data from nurses and midwives on different variables.

### 3.9. Data Collectors and Data Collection Procedures

Ten diploma midwives and nurses collected data. Five supervisors who have a first degree in midwifery/nursing with experience in data collection were involved in supervision. Training was given for data collectors and supervisors before the pretest.

### 3.10. Data Quality Control

Data quality was controlled through training of data collectors on objectives, questionnaire, and ways of administering questionnaire. And also, pretest was conducted in 5% of the sample size in Sodo Christian Hospital and Dubo Hospital, and then the necessary arrangements and corrections were made. All filled questionnaires were checked for completeness, accuracy, and consistency. Strong supervision was carried out by the supervisors and principal investigator throughout the data collection period.

### 3.11. Data Processing and Analysis

The data were entered to Epi data version 3.02 and exported to the SPSS statistical package version 22 for further analysis. Data were presented in frequency, proportions, and summary statistics to describe the study variables and factors under the study. Dependent variable was computed from 22 knowledge questions. Then, mean was calculated, and those above and equal to mean were considered as having a good knowledge. Bivariate and multivariate analyses were carried out to identify variables that were significantly associated with knowledge of immediate new born care. Hosmer-Lemeshow and Omnibus tests were performed to test for model fitness.

Variables whose *p* value was ≤0.25 in bivariate analysis became candidates for multivariable logistic regression. Finally, multivariate analysis was performed to investigate independent factors by controlling for possible confounders. Variables were interpreted as having statistically significant association when *p* < 0.05 and AOR at 95% CI were used to identify strength of association between independent and dependent variables in multivariable analysis.

### 3.12. Ethical Consideration

Ethical approval letter was taken from research review committee of Arba Minch University, College of Medicine and Health Sciences. An official letter from the university was submitted to Wolaita Zone health department. Then, official letters were written from Wolaita Zone health department to respective Woreda Health Office and health facilities. Data were collected after taking informed written consent from each participating nurse and midwives at their free time.

## 4. Result

### 4.1. Sociodemographic Characteristics

From the total of 218 nurses and midwives working in obstetric care units of selected health facilities, 216 nurses and midwives participated in the study making a response rate of 99.1%. Out of 216 participants in the study, 174 (80.6%) were females. The median age of respondents was 26. Regarding profession, 163 (75.5%) of the participants were midwives and 53 (24.5%) were nurses. Regarding their educational qualification, 152 (70.4%) participants have a diploma level as shown in Table 1.

### 4.2. Facility- and Personnel-Related Characteristics

Regarding work experience of study participants, 161 (74.5%) had less than five years work experience, and participants who had greater than six years of work experience in labor and delivery unit were 55 (25.5%). Among the study participants, 43 (19.9%) responded that they had no interest to work in delivery room. 148 (68.5%), 133 (61.6%), and 128 (59.3%) of the participants responded the presence of guidelines on immediate new born care, equipment for newborn care, and vaccines and drugs for newborn care at their health institution, respectively. Regarding training, 147 (68.1%) of the participants got training on ENC as described in Table 2.

### 4.3. Knowledge of Hypothermia and Thermal Protection

Regarding hypothermia protection by placing newborn immediately after birth, majority responded that the newborn should be kept on the mother's chest/belly immediately after birth. Concerning time of bathing, 68 (31.4%) of the respondents did not know the recommended time when newborn should be bathed after delivery.

### 4.4. Knowledge of Airway Clearance and Neonatal Resuscitation

Concerning measures to be taken if babies are not crying immediately after delivery, 153 (70.8%) responded that the correct measure is calling a help and start resuscitation, 36 (16.7%) responded covering the baby and allowing skin-to-skin contact, and the remaining 27 (12.5%) responded putting the baby on newborn table and give mother care.

Regarding the position of the baby's head to open the airway, 137 (63.4%) of the respondents responded that the baby's head should be slightly extended, and the remaining participants did not able to respond the correct position of the head; 59 (27.3%) responded as flexed position of the head, and 20 (9.3%) of them responded as hyperextended position of the head as shown in Table 3.

### 4.5. Knowledge of Breastfeeding

**Figure 1:**
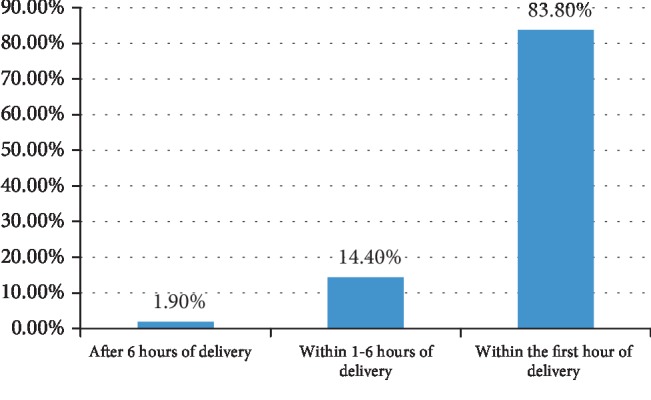
Knowledge of midwives and nurses of the time of initiation of breastfeeding immediately to born babies at Wolaita Zone, Southern Ethiopia, March-April 2019.

Regarding breastfeeding, majority of the participants responded that initiation of breastfeeding after delivery should be taken within the first hour of delivery as shown in Figure 1. When the participants were asked about the duration of exclusive breastfeeding, 172 (79.6%) respondents said that the mother should feed her baby exclusively for the first six months, 3 (1.4%) responded exclusive breastfeeding for less than six months, and 41 (19%) responded mother should feed exclusively for more than six months.

### 4.6. Knowledge of Prevention of Infection of Cord and Eye

From the respondents, half of them, 109 (50.5%) responded that the cord of a crying baby should be clamped or tied at 2-3 minutes of delivery or after the pulsation of umbilical artery stopped and 45 (20.8%) and 62 (28.7%) responded that the cord should be clamped or tied immediately after delivery and within 1-2 minutes of delivery, respectively. Out of the total respondents, the majority, 182 (84.2%) responded using sterile scissor to cut the cord, and 14 (6.5%) responded that new surgical blade can be used to cut the cord whereas the other 20 (9.3%) responded using clean scissor to cut the cord. The majority, 196 (90.7%), responded that silver nitrate/tetracycline can be applied for the treatment of eye infection for newborn, and only 13 (6%) responded that sterile water can be used to clean infected eyes as shown in Table 4.

### 4.7. Knowledge of Care of Babies with Their Respective Birth Weight

**Figure 2:**
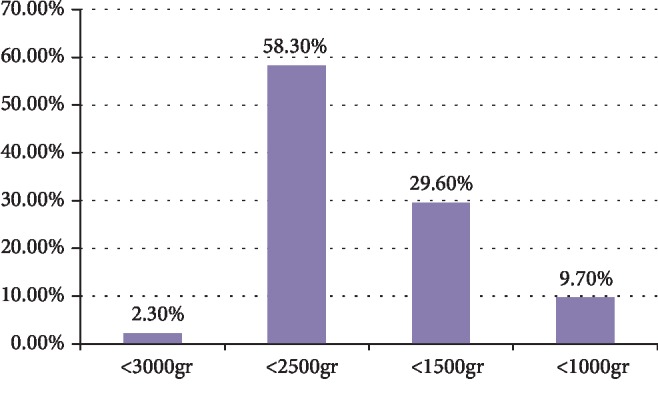
Knowledge of midwives and nurses on the classification of low birth-weight for newborn, March-April 2019.

About the recommended cares of low birth weight babies, 99 (45.8%), 137 (63.4%), 27 (12.5%), and 8 (3.7%) of the participants responded breastfeeding early and frequently, keeping the child warm, infection prevention, and bathing often, respectively, as care for low birth-weight babies. Participants who had knowledge of prevention of newborn bleeding were 200 (92.6%), and 109 (50.5%) had knowledge about the correct dose of vitamin K to be given to a full-term newborn as shown in Figure 2.

### 4.8. Provider's Knowledge of Immediate Newborn Care

**Figure 3:**
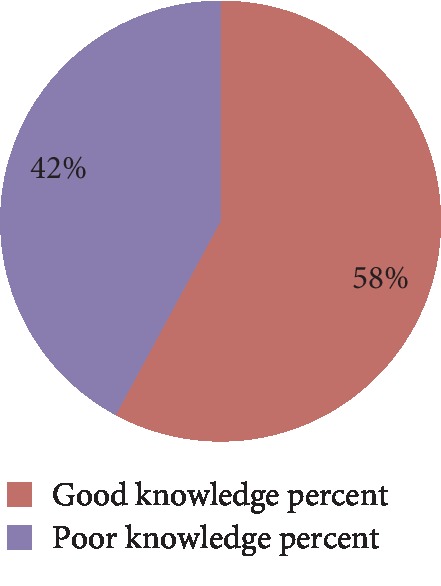
Participant's knowledge of essential newborn care practice in Wolaita Zone, March-April 2019.

Knowledge of essential newborn care was assessed with 22 knowledge-based questions. Then, the mean was calculated. The overall knowledge of nurse and midwife on immediate newborn care in public health facilities of Wolaita Zone was 58% at 95% CI (51%, 65%) as shown in Figure 3.

### 4.9. Factors Associated with Knowledge of Immediate Newborn Care

Monthly income, sex, type of profession, education status, work experience in delivery unit, type of facility, interest to work in delivery unit, got in-service training, availability of BEmONC guidelines, availability of equipment, and availability of drug and vaccines were variables with *p* value less than or equal to 0.25 in bivariate analysis.

Those variables with *p* value ≤ 0.25 in bivariate analysis became candidates for multivariable analysis. Among those variables entered in multivariable analysis, the type of profession, education qualification, interest to work in delivery unit, and the presence of BEmONC guideline were identified to be significantly associated with knowledge of immediate new born care.

The type of profession was significantly associated with participants' knowledge of essential newborn care. Midwives were 5.79 times more knowledgeable about essential newborn care than nurses (AOR 5.79 [CI: 2.47, 13.58]). Educational qualification had statistically significant association with knowledge of ENC (AOR 3.26 [CI: 1.42, 7.52]).

Interest to work in delivery room had statistically significant association with knowledge of ENC (AOR 4.85 [CI: 1.89, 12.42]). The presence of national BEmONC guideline had statistically significant association with knowledge of ENC (AOR 2.29 [CI: 1.18, 4.45]) as shown in Table 5.

## 5. Discussion

This study was planned to determine the magnitude of knowledge of ENC and to identify the associated factors on immediate newborn care among nurse and midwife in public health facilities of Wolaita Zone. The knowledge of nurses and midwives in the study area was lower than what is expected initially.

The knowledge of nurses and midwives on immediate newborn care was 57.9% (95% CI [51%, 65%]). This finding is in line with the study conducted in Jimma zone which was (52.2%) [3] and Bahir Dar city (56%) [2]. This similarity may be due to the similarity in the study population and might be due to similar access to in-service training on essential new born care. This finding was higher than the study conducted in Uganda (46.5%) [9] and Sudan Khartoum (50.6%) [12]. However, this finding was lower than the study conducted in Tigray eastern zone (74.65%) [19, 20]. The difference might be due to the difference in sample size, study setting, and study period and also may be due to the difference in educational qualification across regions.

Concerning factors associated with knowledge of essential new born care (finding from this study shows the type of profession, educational qualification, presence of BEmONC guideline, and interest to work in delivery room) were identified to be significantly associated with knowledge of immediate new born care.

Regarding the type of profession, midwives were more knowledgeable than nurses on essential newborn care. This study finding is similar with that of study conducted in Jimma zone. This similarity may be due to the similarity in study participants. However, this finding was contrary to the study conducted in Kaplivastu District, Nepal, which reports that there is no statistical difference in the level of knowledge among nurses and midwives [3, 21]. The difference might be due to the difference in the study sample size, in health policy, and in study period.

Regarding educational qualification, those with bachelor level of education were more knowledgeable than diploma holders on ENC which is similar with that of study conducted in Jimma zone which shows diploma holders were less knowledgeable than bachelor degree holders on essential new born care components [3]. This similarity might be due to similarity in healthcare managing system across the country which makes composition of professionals in delivery unit is similar.

Professionals who were interested to work in the delivery room were nearly five times more knowledgeable than those who had no interest which is similar with that of study conducted in Jimma zone [3]. This might be due the fact that people get more information and knowledge on areas where they are interested to know and practice. Nurses and midwives who work in health facilities where BEmONC guidelines were available were two times more knowledgeable of essential newborn care. However, there was no study which support or contradict this finding. But this might give some information on need of provision of guideline in addition to providing training for professionals.

## 6. Conclusion and Recommendation

The knowledge of midwives and nurses on essential newborn care practice was inadequate in the study area. A low level of knowledge was identified in ways of thermal protection, newborn resuscitation, cord care, weighing the newborn, defining LBW, the recommended care for low birth-weight baby, and the recommended dose of vitamin K for full-term baby take most share of the knowledge gap of respondents. Types of profession, education qualification, interest to work in delivery unit, and the presence of BEmONC guideline in delivery unit were independent factors which were associated with knowledge of immediate newborn care.

Based on the study findings, we forwarded the following recommendations to respective institutions and bodies:

- Delivery unit head should follow staff while they attend delivery, then document for further identification of problems associated with the gap on knowledge of immediate newborn care
- The managing bodies of hospitals and health center should strive for better knowledge through providing a chance for continuous education and making supportive supervision on staff in the delivery room and motivating staff to work best with interest
- Zonal health department should recruit more midwives, provide BEmONC manuals, train midwives and nurses, and monitor for proper implementation of training contents while they are applying their knowledge

## Figures and Tables

**Table 1:** Sociodemographic characteristics of midwives and nurses working in public health facilities of Wolaita Zone, Southern Ethiopia, March-April 2019.

Variables	Category	Frequency	Percent
Age	20-30	196	90.7
	31-40	20	9.3

Sex	Male	42	19.4
	Female	174	80.6

Marital status	Never married	66	30.5
	Married	149	69
	Widowed	1	0.5

Profession	Nurse	53	24.5
	Midwife	163	75.5

Educational qualification	Diploma	152	70.4
	Degree	64	29.6

Monthly income in Ethiopian birr	2,000-2,999	42	19.4
	3,000-3,999	103	47.7
	4,000-4,999	47	21.8
	>5,000	24	11.1

**Table 2:** Facility- and personnel-related characteristics of midwives and nurses working in public health facilities of Wolaita Zone, Southern Ethiopia, March-April 2019.

Variables	Category	Frequency	Percent
Type of facility	Hospital	54	25
	Health center	162	75

Got in-service training	Yes	147	68.1
	No	69	31.9

Experience in delivery (in year)	<6	161	74.5
	≥6	55	25.5

Interest to work in delivery unit	Yes	173	80.1
	No	43	19.9

Presence of guideline	Yes	133	61.6
	No	83	38.4

Availability of equipment	Yes	148	68.5
	No	68	31.5

Availability of drug and vaccines	Yes	128	59.3
	No	88	40.7

**Table 3:** Knowledge on airway clearance and neonatal resuscitation among midwives and nurses working in public health facilities of Wolayta Zone, Southern Ethiopia, March-April, 2019.

Variables	Response	Frequency	Percent
Time of newborn care start	Before birth	17	7.9
	During birth	45	20.8
	After birth	154	71.3

Place the newborn was kept immediately after birth	Beside the mother	34	15.7
	With someone else	3	1.4
	On the mother's chest/belly	142	65.7
	On newborn bed/table	37	17.1

Measures to be taken if the baby is not breathing well after drying, clearing the air way, and rubbing the back once or twice	More stimulation to breathe	62	28.7
	Ventilation with bag and mask	154	71.3

The mentioned breaths/min	30 breaths per minute	68	31.5
	40 breaths per minutes	98	45.4
	60 breaths per minute	50	23.1

Prevention of eye infections after delivery	Apply nothing	4	1.9
	Apply breastmilk on the eye	3	1.4
	Clean eye with sterile water	13	6
	Apply silver nitrate/tetracycline	196	90.7

**Table 4:** Knowledge related responses on ENC among midwives and nurses working in public health facilities of Wolayta Zone, Southern Ethiopia, March-April, 2019.

Variables	Response	Frequency	Percent
Time of newborn care start	Before birth	17	7.9
	During birth	45	20.8
	After birth	154	71.3

Place the newborn was kept immediately after birth	Beside the mother	34	15.7
	With someone else	3	1.4
	On the mother's chest/belly	142	65.7
	On newborn bed/table	37	17.1

Measures to be taken if the baby is not breathing well after drying, clearing the air way, and rubbing the back once or twice	More stimulation to breathe	62	28.7
	Ventilation with bag and mask	154	71.3

The mentioned breaths/min	30 breaths per minute	68	31.5
	40 breaths per minutes	98	45.4
	60 breaths per minute	50	23.1

Prevention of eye infections after delivery	Apply nothing	4	1.9
	Apply breastmilk on the eye	3	1.4
	Clean eye with sterile water	13	6
	Apply silver nitrate/tetracycline	196	90.7

**Table 5:** Bivariate and multivariate analysis result of factors associated with knowledge of essential newborn care among midwives and nurses working in public health facilities of Wolaita Zone, Southern Ethiopia, March-April 2019.

Independent variables	Category	Knowledge of ENC		COR [95% CI]	AOR [95% CI]	*p* value
		Good	Poor			
Type of profession	Midwife	112	51	0.88 [0.35, 2.22]	5.79 [2.47, 13.58]	0.001^∗∗^
	Nurse	13	40	1	1	

Educational qualification	Bachelor	48	16	2.92 [1.53, 5.59]	3.26 [1.42, 7.52]	0.006^∗∗^
	Diploma	77	75	1	1	

Type of facility	H/C	86	82	0.24 [0.11, 0.53]	0.51 [0.21, 1.23]	0.13
	Hospital	39	9	1	1	

Interest to work in delivery unit	Yes	114	59	0.18 [0.08, 0.38]	4.85 [1.89, 12.42]	0.001^∗∗^
	No	11	32	1	1	1

Presence of guideline	Yes	91	42	0.32 [0.18, 0.57]	2.29 [1.18, 4.45]	0.014^∗∗^
	No	34	49	1	1	1

Availability of equipment	Yes	95	53	0.44 [0.25, 0.79]	1.41 [0.69, 2.87]	0.35
	No	30	38	1	1	1

Vaccine and drug availability	Yes	81	47	0.58 [0.33, 10]	1.24 [0.59, 2.61]	0.56
	No	44	44	1	1	1

Got training	Yes	97	50	0.35 [0.19, 0.64]	1.43 [0.65, 3.13]	0.37
	No	28	41	1	1	

Monthly income (ETB)	>5,000	18	6	3.00 [0.99, 9.05]	0.84 [0.16, 4.52]	0.84
	4,000-4,999	31	16	1.94 [0.83, 4.55]	0.50 [0.15, 1.69]	0.27
	3,000-3,999	55	48	1.15 [0.56, 2.35]	0.58 [0.22, 1.55]	0.27
	2,000-2,999	21	21	1	1	

Work experience in delivery unit	>6 years	38	17	1.90 [0.99, 3.64]	1.127 [0.518, 2.45]	0.76
	0-5 years	87	74	1	1	

Sex	Male	29	13	1.81 [0.88, 3.72]	2.82 [0.994, 8.025]	0.051
	Female	96	78	1	1	

^∗∗^Statistical significant association in AOR.

## Data Availability

Data will be available on request to Zerihun Zana (zerihun7777@gmail.com) or Aseb Arba (40aseb@gmail.com).

## References

[1] World Health Organization (2017). *WHO Recommendations on Newborn Health: Guidelines Approved by the WHO Guidelines Review Committee*.

[2] Yemaneh Y., Dagnachew E. (2017). Knowledge and practice of immediate new born care (inc.) among health professionals in governmental health facilities of Bahir Dar City, North Ethiopia 2016. *Quality in Primary Care*.

[3] Negussie B. B., Hailu F. B., Megenta A. D. (2017). Knowledge and practice of essential newborn care and associated factors among nurses and midwives working at health centers in Jimma Zone, Ethiopia, 2016. *Journal of Nursing & Care*.

[4] Worku B., Gessessie M. (2012). Newborn care training manual, essential new born care for every baby. *Federal Ministry of Health Ethiopia EPS*.

[5] Paul V. (2014). *Newborn Nursing for Facility Based Care. Learner’s Guide*.

[6] Bashir I., Migiro S. (2004). *National Guidelines for Quality Obstetrics and Perinatal Care*.

[7] World Health Organization (2018). *Newborns: Reducing Mortality*.

[8] Lerberghe W. V. M. A., Matthews Z., Wolfheim C. (2005). *Make Every Mother and Child Count*.

[9] Waiswa P., Kallander K., Peterson S., Tomson G., Pariyo G. W. (2010). Using the three delays model to understand why newborn babies die in eastern Uganda. *Tropical Medicine & International Health*.

[10] Malhotra S., Zodpey S. P., Vidyasagaran A. L. (2014). Assessment of essential newborn care services in secondary-level facilities from two districts of India. *Journal of health, population, and nutrition*.

[11] de Graft-Johnson J., Vesel L., Rosen H. E. (2017). Cross-sectional observational assessment of quality of newborn care immediately after birth in health facilities across six sub-Saharan African countries. *BMJ open*.

[12] Faiza A. N. (2013). Assessment of knowledge, attitude and practices of nurse midwives towards immediate care of the newborn in Khartoum state teaching hospitals. *Journal of American Science*.

[13] Traoré F. D., Sylla M., Diall H. (2018). Knowledge of health professionals on essential newborn care in Bamako, Mali. *Open Journal of Pediatrics*.

[14] Getachew A., Ricca J., Cantor D. (2011). *Quality of Care for Prevention and Management of Common Maternal and Newborn Complications: A Study of Ethiopia’s Hospitals*.

[15] Millar tw K., MHTF (2014). Lancet launches every newborn series: where we have been and where we need to go. *Lancet*.

[16] EPHI (2016). *Ethiopian Emergency Obstetric and Newborn Care (EmONC) Assessment*.

[17] Central Statistical Agency (CSA) [Ethiopia] (2016). *Ethiopia Demographic and Health Survey 2016*.

[18] Bereka B. (2016). *Assessment of Knowledge and Practice of Essential Newborn Care and Associated Factors among Nurses and Midwives Working at Health Centers in Jimma Zone, Oromia Regional State, South West of Ethiopia, 2016*.

[19] Berhe A. K., Tinsae F., Gebreegziabher G. (2017). Knowledge and practice of immediate newborn care among health care providers in eastern zone public health facilities, Tigray, Ethiopia, 2016. *BMC Pediatrics*.

[20] Ayiasi R. M., Criel B., Orach C. G., Nabiwemba E., Kolsteren P. (2014). Primary healthcare worker knowledge related to prenatal and immediate newborn care: a cross sectional study in Masindi, Uganda. *BMC Health Services Research*.

[21] Acharya D., Paudel R., Gautam K., Gautam S., Upadhyaya T. (2016). Knowledge of maternal and newborn care among primary level health workers in Kapilvastu district of Nepal. *Annals of Medical and Health Sciences Research*.

